# *Granulibacter bethesdensis*, a Pathogen from Patients with Chronic Granulomatous Disease, Produces a Penta-Acylated Hypostimulatory Glycero-D-talo-oct-2-ulosonic Acid–Lipid A Glycolipid (Ko-Lipid A)

**DOI:** 10.3390/ijms22073303

**Published:** 2021-03-24

**Authors:** Artur Muszyński, Kol A. Zarember, Christian Heiss, Joseph Shiloach, Lars J. Berg, John Audley, Arina Kozyr, David E. Greenberg, Steven M. Holland, Harry L. Malech, Parastoo Azadi, Russell W. Carlson, John I. Gallin

**Affiliations:** 1 Complex Carbohydrate Research Center, University of Georgia, Athens, GA 30602, USA; cheiss@ccrc.uga.edu (C.H.); azadi@ccrc.uga.edu (P.A.); rcarlson@ccrc.uga.edu (R.W.C.); 2 Laboratory of Clinical Immunology and Microbiology, National Institute of Allergy and Infectious Diseases, National Institutes of Health, Bethesda, MD 20892, USA; kzarember@niaid.nih.gov (K.A.Z.); lberg@wesleyan.edu (L.J.B.); audleyj2@gmail.com (J.A.); arina.kozyr@nih.gov (A.K.); david.greenberg@utsouthwestern.edu (D.E.G.); sholland@niaid.nih.gov (S.M.H.); hmalech@niaid.nih.gov (H.L.M.); 3 Biotechnology Core, National Institute of Diabetes and Digestive and Kidney Diseases, Bethesda, MD 20892, USA; ljs@helix.nih.gov

**Keywords:** lipopolysaccharide, lipid A, Gram-negative pathogen, immunodeficiency

## Abstract

*Granulibacter bethesdensis* can infect patients with chronic granulomatous disease, an immunodeficiency caused by reduced phagocyte NADPH oxidase function. Intact *G. bethesdensis* (*Gb*) is hypostimulatory compared to *Escherichia coli*, i.e., cytokine production in human blood requires 10–100 times more *G. bethesdensis* CFU/mL than *E. coli*. To better understand the pathogenicity of *G. bethesdensis*, we isolated its lipopolysaccharide (*Gb*LPS) and characterized its lipid A. Unlike with typical *Enterobacteriaceae*, the release of presumptive Gb lipid A from its LPS required a strong acid. NMR and mass spectrometry demonstrated that the carbohydrate portion of the isolated glycolipid consists of α-Man*p*-(1→4)-β-Glc*p*N3N-(1→6)-α-Glc*p*N-(1⇿1)-α-Glc*p*A tetra-saccharide substituted with five acyl chains: the amide-linked N-3′ 14:0(3-OH), N-2′ 16:0(3-O16:0), and N-2 18:0(3-OH) and the ester-linked O-3 14:0(3-OH) and 16:0. The identification of glycero-d-talo-oct-2-ulosonic acid (Ko) as the first constituent of the core region of the LPS that is covalently attached to GlcpN3N of the lipid backbone may account for the acid resistance of *Gb*LPS. In addition, the presence of Ko and only five acyl chains may explain the >10-fold lower proinflammatory potency of *Gb*Ko–lipidA compared to *E. coli* lipid A, as measured by cytokine induction in human blood. These unusual structural properties of the *G.bethesdensis* Ko–lipid A glycolipid likely contribute to immune evasion during pathogenesis and resistance to antimicrobial peptides.

## 1. Introduction

Bacteria belonging to *Acetobacteraceae*, the Gram-negative family associated with production of acetic acid from ethanol, occur commonly in the environment and are generally considered non-pathogenic in humans. However, over the past decade, at least six genera within this family have been recognized as pathogens in human subjects with genetic immunodeficiencies, with intravenous access devices, or with a history of intravenous drug use [1,2,3,4,5,6]. Among these emerging pathogens is *Granulibacter bethesdensis*, non-motile, catalase-positive, and oxidase-negative coccobacillus first described in 2006 [7], which, to date, has only been isolated from human patients with chronic granulomatous disease (CGD), a rare primary immunodeficiency caused by deleterious mutations in the phagocyte NADPH oxidase (NOX2). Typically, NOX2 produces the superoxide anion that, together with nitric oxide, can generate antimicrobial peroxinitrite or be used by superoxide dismutase to form antimicrobial hydrogen peroxide that acts directly on microbes or can be used by myeloperoxidase to form antimicrobial hypohalous acids (bleach) and chlorine [8]. As a consequence of reduced NOX2 function, patients with CGD suffer from frequent opportunistic and often deadly infections by *Aspergillus*, *Staphylococcus*, *Burkholderia*, and other microbes.

*G. bethesdensis* was a previously undescribed genus and species first isolated from a patient with CGD in 2003 [4,7]. Since its description, at least 10 individuals with CGD have been diagnosed with *G. bethesdensis* infection worldwide, and 3 cases have been fatal [2,9]. *G. bethesdensis* can cause lymphadenitis, bacteremia, and meningitis and is the only *Acetobacteraceae* member for which Koch’s postulates have been fulfilled in mammals [4]. Serology suggests that *G. bethesdensis* may be under-reported in CGD and that long-term, clinically latent infections may also occur [10].

Despite its recent description, *G. bethesdensis* remains the best-studied member of the *Acetobacteraceae* family in terms of microbial pathogenesis. Although killed by neutrophils, monocytes, and monocyte-derived macrophages from healthy subjects, *G. bethesdensis* is resistant to attack by these cell types from NOX2-defective CGD patients [11,12]. Efficient internalization of *G. bethesdensis* by neutrophils requires complement, but it was noted that on a per cell basis, 10–100 times more *G. bethesdensis* is required than *E. coli* to induce a respiratory burst in normal neutrophils [12]. Furthermore, *G. bethesdensis* was less effective at inducing cytokine production in monocytes than other Gram-negative bacteria [13], suggesting that it either lacks molecular patterns that typically activate the immune system or that these patterns, e.g., lipopolysaccharides (LPS), are hidden by capsular polysaccharides or other materials.

In contrast to what is known about enterobacterial LPS and lipid A, relatively little is known about the properties of these molecules in *Acetobacteraceae*. The LPS of a soil isolate, *Acidiphilium* GS18h/ATCC55963, contains glucosamine and Kdo but was non-toxic compared to the *E. coli* LPS in mice [14] and induced lower cytokine concentrations compared to the *E. coli* LPS in mouse and human monocytes [15]. The LPS from *Acetobacter aceti* was partially purified and was nearly 1000-fold less potent than the LPS from *Pantoea* (formerly *Enterobacter*) *agglomerans*, an opportunistic pathogen [16]. *Acetobacter xylinum*, a cellulose-producing bacterium, possesses an LPS containing Kdo but was not analyzed immunologically [17]. Recently, in two independent studies, it was shown that *Acetobacter pasteurianus* NBRC3283 and *A. pasteurianus* CIP103108 produce a hexa-acylated lipid A with a Ko (D-gylcero-D-talo-oct-2-ulosonic acid) substitution [18,19]. To better understand how *G. bethesdensis* avoids activation of immune cells and is resistant to certain antimicrobial peptides, we purified and characterized the structure and bioactivity of the lipid A from this organism.

## 2. Results

### 2.1. Hypostimulatory Effect of G. bethesdensis on Human Blood Cells

**Figure 1:**
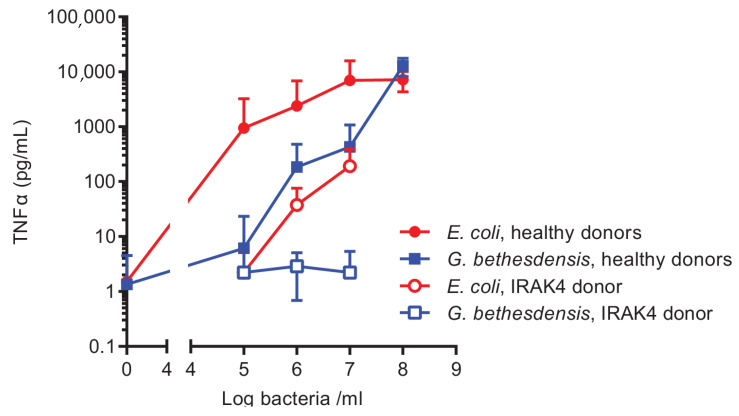
TNFα induction in human whole blood following incubation with heat-killed *Granulibacter bethesdensis* or *Escherichia coli*. Anticoagulated whole blood was incubated for 6 h with bacteria, as described in the Materials and Methods section, and supernatants were assayed for TNFα by ELISA. The Mean + SD is shown for 13–15 healthy donors and a single IRAK4-deficient patient assayed on two separate days.

Both viable and heat-killed *G. bethesdensis* were previously reported to be at least 10–100 times less potent at stimulating the neutrophil respiratory burst than equivalent numbers of *E. coli* [12]. Here, we used an ex vivo whole-blood assay of cytokine production to confirm the relative stimulatory potency of *E. coli* and *G. bethesdensis* in an independent experimental setting. Autoclaved bacteria were used so that precise enumeration of bacterial colony forming units (CFUs) could be accomplished in advance. As shown in Figure 1, *E. coli* TOP10 induced a dose-dependent increase in the production of tumor necrosis factor alpha (TNFα) that plateaued at about 1 × 10^7^ CFU equivalents/mL. In contrast, between 10–100 times greater numbers of *G. bethesdensis* cells/mL were required to achieve similar cytokine accumulation levels at sub-maximum responses. Markedly decreased levels of cytokines were induced by both bacteria in blood from a previously described patient with a genetic deficiency in interleukin-1 receptor associated kinase 4 (IRAK4) whose neutrophils and monocytes had impaired signaling through the Toll-like receptors [20], implicating this signaling intermediate, and possibly Toll-like receptors, in the detection of these bacteria in blood. 

### 2.2. Characterization of the G. bethesdensis LPS

**Figure 2:**
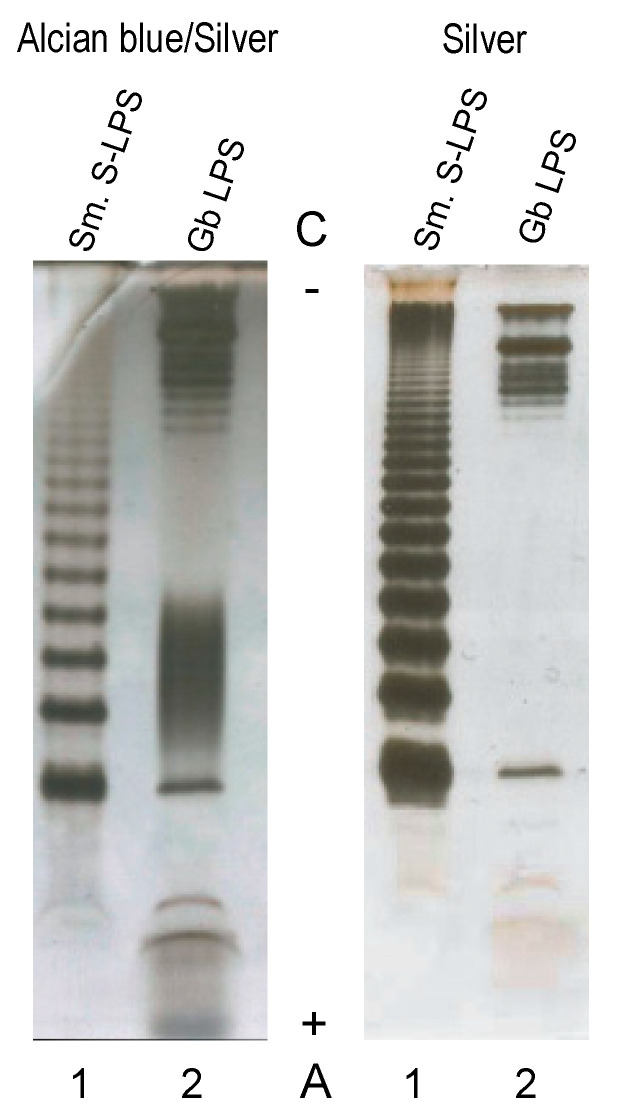
Deoxycholic acid (DOC)-PAGE analysis of a lipopolysaccharide (LPS) isolated from *G. bethesdensis*. Samples were stained with Alcian blue, followed by silver staining, or only with silver. (Lane 1) *Salmonella enterica* sv. Minnesota, S-type LPS (Sm. S-LPS) (1 µg). (Lane 2) *G. bethesdensis* LPS (Gb LPS) (1 µg). (A+) anode and (C^−^) cathode regions of the PAGE gel.

We hypothesized that the relatively poor activation of human immune cells by *Granulibacter* might be due to an unusual lipopolysaccharide (LPS). To directly test whether *Granulibacter* LPS (*Gb*LPS) resembles that of typical Gram-negative bacteria such as *E. coli*, washed bacterial cells were extracted using the hot phenol-water extraction method of Westphal and Jann [21]. The aqueous layer contained the most material, and compositional analyses of both supernatant and pellet after ultracentrifugation revealed essentially identical profiles containing predominantly Rha, Glc, Xyl, Hep, and fatty acyl components consisting of 14:0(3-OH), 16:0(3-OH), 18:0(3-OH) hydroxyl and 16:0, 18:0, and 18:1 straight-chain fatty acids. No Kdo was detected. Polyacrylamide gel electrophoresis in the presence of deoxycholic acid (DOC-PAGE) and silver staining of the water-phase fraction, with or without Alcian blue, revealed the presence of two slower-migrating, prominent bands on the cathode side of the gel, followed by a ladder typical of a smooth LPS (Figure 2). In contrast, staining with Alcian blue prior to sample oxidation and silver staining revealed not only top bands (consistent with silver staining) but also faster-migrating species on the anode side of the gel, suggesting that *Gb* also synthesizes other glycolipids or polysaccharides.

Comparisons of toll-like receptor 4 (TLR4) activation by different smooth LPS structures based on the measured mass are complicated because of the variable amounts of O-polysaccharide per mole of the lipid A moiety. Our approach to overcome this challenge and to compare potencies of lipid A from different fractions and species employs a kinetic *Limulus* hemocyte lysate (LAL) assay, which primarily detects lipid A. The bioactive lipid A content of the various LPS preparations examined were then expressed relative to the World Health Organization standard endotoxin (*E. coli* O113:H10 control standard endotoxin, Associates of Cape Cod). Although the LAL can also be activated by (1→3)-ß-d-glucan, this pathway can be inhibited by use of Glucashield buffer (Associates of Cape Cod). Using this approach, we measured LAL activity and found that most of the activity is in the supernatant of the aqueous layer, although significant amounts remained in a loose, gel-like LPS pellet. Immunoblotting of *Gb*LPS using monoclonal antibodies (mAb) that readily detected *E. coli* lipid A, mAb 26-5 and mAb 222-5, were negative, suggesting that *Gb*LPS lacks the specific epitopes targeted by these antibodies, i.e., enterobacterial lipid A and an LPS core, respectively (data not shown). For that reason, we undertook to determine the structure of lipid A.

### 2.3. Chemical and Structural Characterization of Ko-Containing Lipid A Glycosyl Substituents

**Figure 3:**
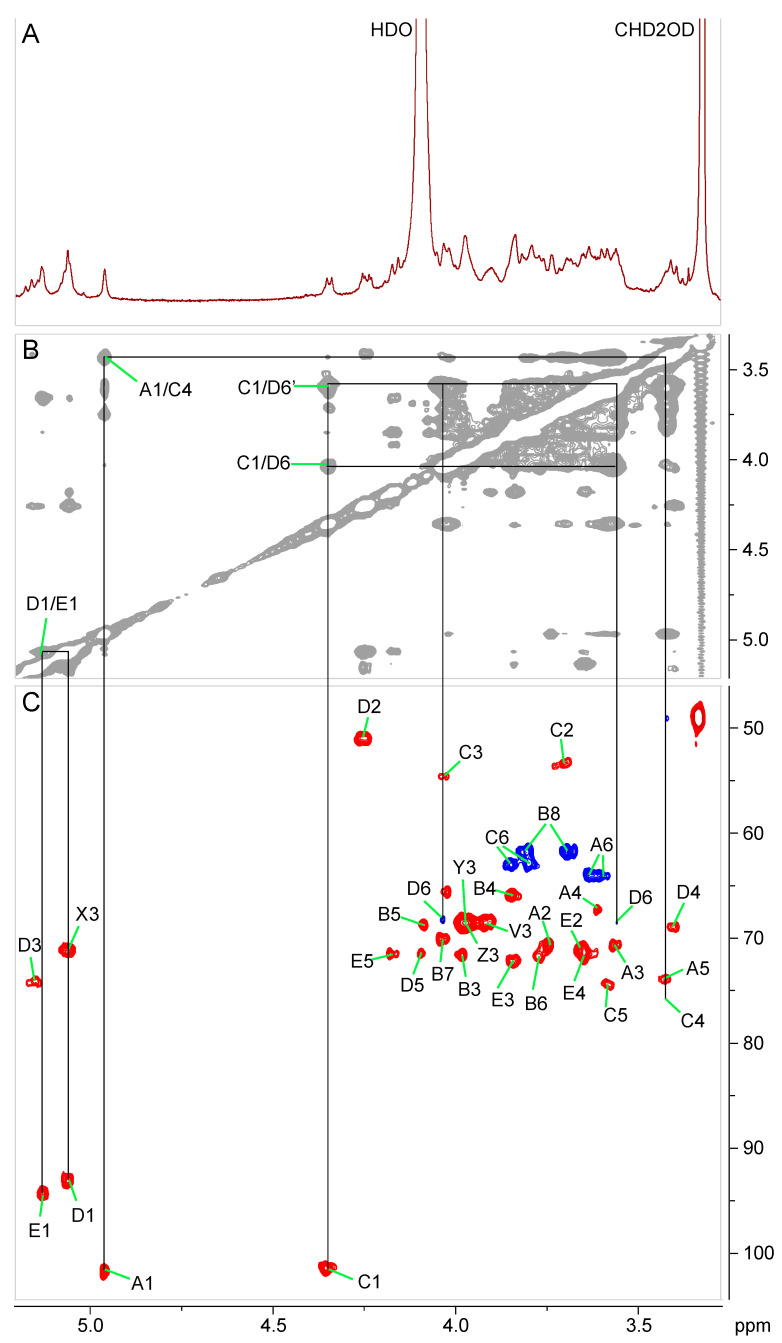
Partial ^1^H (**A**), ^1^H-^1^H nuclear Overhauser effect spectroscopy (NOESY) (**B**), and ^1^H-^13^C-heteronuclear single-quantum correlation spectroscopy (HSQC) (**C**) NMR spectra of *G. bethesdensis* Ko–lipid A in 3:1 CDCl_3_–CD_3_OD solution. The spectra show the region containing mostly carbohydrate signals. The labels are explained in Table 1 and Table 2.

**Figure 4:**
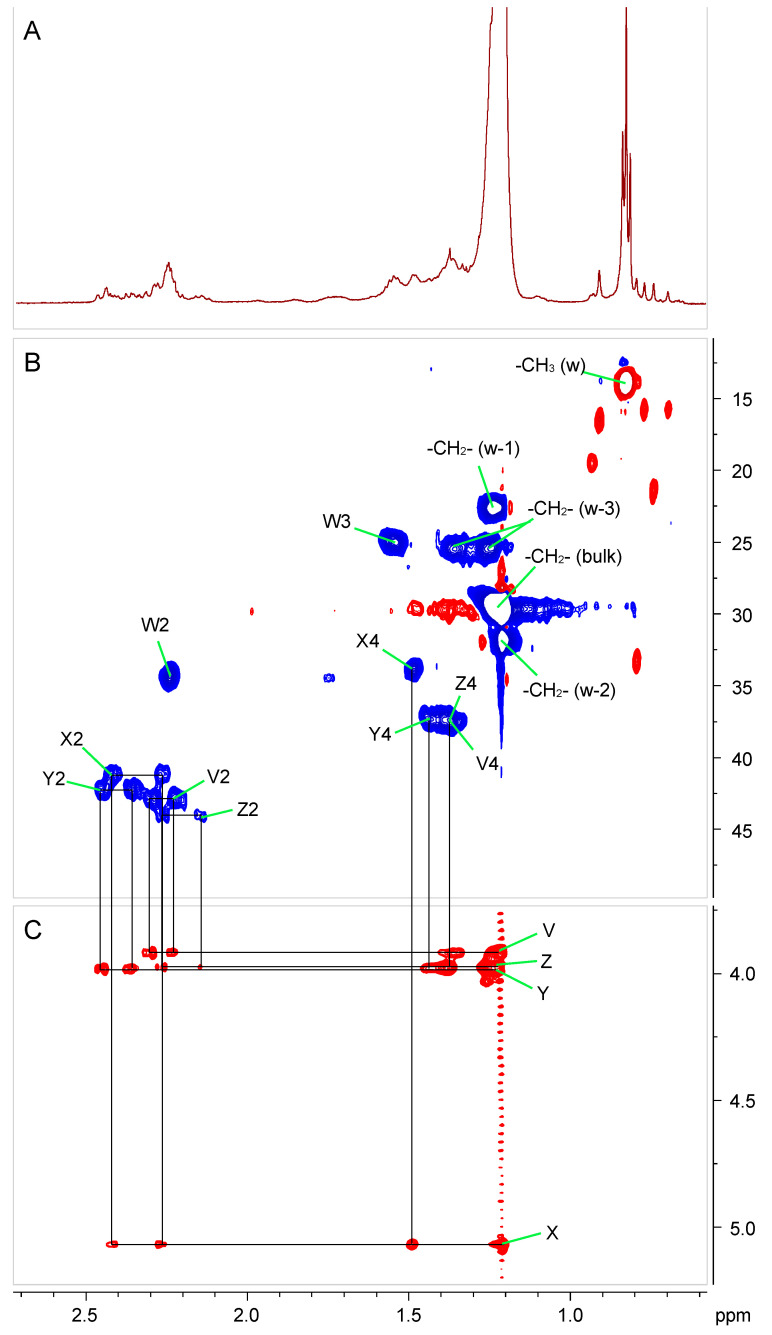
Partial ^1^H (**A**), ^1^H-^13^C-HSQC (**B**), and ^1^H-^1^H total correlation spectroscopy (TOCSY) (**C**) NMR spectra of *G. bethesdensis* Ko–lipid A in 3:1 CDCl_3_–CD_3_OD solution. The spectra show the region containing most fatty acid signals. The labels are explained in Table 2.

Initially, we attempted to liberate lipid A from the LPS by using 1% acetic acid (HOAc) hydrolysis at 102 °C, but this yielded only trace amounts of lipid A, even after 6 h of hydrolysis. In addition, we detected contaminating glycosyl residues likely originating from the polysaccharide part of the LPS (not shown). *G. bethesdensis* belongs to *Acetobacteraceae*, the microbial family including bacteria that produce acetic acid from ethanol. Some members of this family, such as *Acetobacter*, have been shown to synthesize d-*glycero*-d-*talo*-oct-2-ulosonic acid (Ko) instead of 3-deoxy-d-*manno*-oct-2-ulosonic acid (Kdo)—a common core region constituent directly attaching to lipid A in the LPS of many Gram-negative bacteria [18]. Acid resistance of Ko has been well documented [22,23], and therefore, lipid A release was attempted under more acidic conditions, in the presence of 0.1 M HCl at 100–102 °C for 4.5 h. Although this harsher hydrolysis condition resulted in partial chemical degradation of the polysaccharide portion of the LPS, it substantially increased the yield of lipid A without changing its major matrix assisted laser desorption/ionization- time of flight mass spectrometry (MALDI-TOF MS) signals from those observed after mild 1% HOAc hydrolysis. The major lipid A species seen under both conditions in the negative ionization mode had an [M-H] mass *m*/*z* of 2140. Composition analysis by gas chromatography-mass spectrometry (GC-MS) of trimethylsilyl (TMS) ethers of methyl glycosides of the lipid A released with 0.1 M HCl revealed the presence of Man, GlcA, and glucosamine (GlcN). We also confirmed in the modified glycosyl linkage analysis, with methylation done after carboxyl reduction, that both Man*p* and GlcA*p* were only linked in the anomeric position and thus were terminal residues. In addition, GC-MS analysis of alditol acetates of the lipid A hydrolyzed with 4M HCl for 4 h, followed by peracetylation, detected 2,3-diamino-2,3-dideoxy glucose (GlcN3N). This substituent was identified by characteristic electron impact mass spectrometry (EI-MS) fragments at *m*/*z* 144 (further cleaved to *m*/*z*→102, 103, 84, 85), accompanied with a fragment ion at *m*/*z* 288 (→228, 169, 168, 169), and a weak signal at *m*/*z* 216 (→156, 114), consistent with EI-MS assignments presented elsewhere [24]. The presence of Man, Gal, and GlcA in the sample was further supported by NMR analysis, which additionally confirmed the presence of Ko. The finding of Ko may explain the resistance of the carbohydrate–lipid A bond of the LPS to 0.1 M HCl hydrolysis. The 1D proton NMR spectrum of lipid A, dissolved in 3:1 (*v*/*v*) CDCl_3_–CD_3_OD, showed rather broad signals resulting from aggregation of the glycolipid (Figure 3A). The upfield region included intense signals arising from fatty acid methyl and methylene protons, including a set of α-methylene groups of 3-hydroxy fatty acids between 2.2 and 2.5 ppm (Figure 4A). The downfield region of the proton spectrum showed a set of anomeric peaks around 5 ppm and a complex carbohydrate ring proton region. The 2D correlation spectroscopy (COSY) spectrum revealed that the section around 5 ppm contained, besides anomeric signals characterized by single cross peaks between 3.5 and 4.5 ppm, a signal from the β-proton of an O-acyl-substituted 3-hydroxy fatty acid, as well as at least one proton from an O-acylated sugar position. The most prominent of these, at 5.16 ppm, was correlated indirectly, via a signal at 4.26 ppm, to an anomeric proton at 5.06 ppm (residue D) (Figure 3). The heteronuclear single-quantum correlation spectroscopy (HSQC) spectrum gave corresponding carbon chemical shifts of 93.1, 51.1, and 74.3 ppm for C-1, C-2, and C-3, respectively. These and the remaining proton and carbon chemical shifts in this spin system (Table 1), as well as a one-bond anomeric C-H coupling constant of 169 Hz, together with the monosaccharide composition obtained by GC-MS, identified residue D as 6-linked α-2-N,3-*O*-diacyl-glucosamine. The anomeric signal for a second glycosyl residue resonated at 5.13 ppm. This proton was correlated to four protons in the total correlation spectroscopy (TOCSY) spectrum, the most downfield of which belonged to H-5, with no further cross peaks. This result, together with the proton and carbon chemical shifts of this residue (E) and its one-bond anomeric C-H coupling constant of 177 Hz, were consistent with terminal α-GlcA. A third glycosyl residue (A) had an anomeric proton signal at 4.96 ppm and a one-bond anomeric C-H coupling constant of 170 Hz showing α-configuration. (Figure 3B,C). H-1 showed only a single cross peak in the TOCSY spectrum, suggesting A to be a mannose residue, which was confirmed by the upfield carbon chemical shift of C-4. The remaining assignments of this residue, obtained from inspection of the COSY, TOCSY, and HSQC spectra, were consistent with terminal mannose. The final anomeric signal found in the HSQC spectrum was seen at 4.36 ppm and had a one-bond anomeric C-H coupling constant of 155 Hz, indicative of β-anomeric configuration of this residue (C) (Figure 3). Carbons 2 and 3 resonated at 53.4 and 54.7 ppm, respectively, indicating amino groups in these positions. The TOCSY spectrum showed correlations to five other protons, consistent with *gluco*-configuration. The proton and carbon chemical shifts of C agreed with the structure of β-2,3-diaminoglucose (GlcN3N), in agreement with identification of this residue in the chemical composition.

Because the remaining spin system (residue B) did not include an anomeric proton, its assignment was started with the only remaining unassigned pair of methylene signals in HSQC (3.81, 3.69/61.8 ppm), which were tentatively assigned as H-8a, H-8b/C-8 of Ko and which showed COSY cross peaks to a signal at 4.03 ppm (Ko:H-7). This signal, in turn, was correlated to a peak at 3.77 ppm (Ko:H-6) in COSY. The latter had no further correlations in the COSY spectrum, consistent with the small ^3^J_H5-H6_, due to axial configuration of H-5 in Ko. However, H-6 exhibited a nuclear Overhauser effect spectroscopy (NOESY) cross peak to a signal at 4.09 ppm (Ko:H-5). That signal was correlated to two more signals in the TOCSY spectrum, at 3.98 (Ko:H-4) and 3.85 ppm (Ko:H-3). Due to the extensive aggregation of this glycolipid, NMR relaxation rates were very fast, making it impossible to obtain a useful heteronuclear multiple bond correlation (HMBC) spectrum. Therefore, and because we did not have sufficient material to obtain a 1-D ^13^C-NMR spectrum, the chemical shifts of C-1 and C-2 of the Ko residue, which are not directly attached to protons and therefore were not detected in HSQC, could not be measured. Nevertheless, the proton and other carbon chemical shifts agreed with those published for the identical oligosaccharide structure [18], and thus residue B was identified as α-Ko. Lacking a useful HMBC spectrum, we used a NOESY spectrum to map the connectivity between monosaccharides. Thus, a nuclear Overhauser effect (NOE) contact between H-1 of D and H-1 of E showed that these two residues were connected via a (1↔1)-linkage (Figure 3B). The anomeric proton of residue C showed an inter-residue NOE cross peak to H-6′ of residue D, and the anomeric proton of residue A showed a NOESY correlation with H-4 of residue C. The slight downfield chemical shift displacement of C-6 of residue C indicated substitution at this position, likely by the only remaining glycosyl residue, Ko. Taken together, the data from NMR analysis are consistent with the following pentasaccharide structure for the carbohydrate portion of *G. bethesdensis* Ko–lipid A:
α-Man*p*-(1→4)(α-Ko*p*-(2→6))-β-Glc*p*N3N-(1→6)-α-Glc*p*N-(1↔1)-α-Glc*p*A**A      B       C       D      E**

### 2.4. Fatty Acid Substituents of Ko–Lipid A

The total fatty acid GC-MS composition of Ko–lipid A indicated the presence of 14:0(3-OH) 16:0, 18:0(3-OH), 16:0(3-OH), and 18:1 in approximately a 3:3:2:1:1 ratio. A trace of 18:0 and 19:1 was also detected. *G. bethesdensis* Ko–lipid A is likely a heterogeneous mixture of molecules that vary in their fatty acyl substitution with hydroxyl- and straight-chain (saturated and unsaturated) fatty acids. Furthermore, fatty acid analysis of alkali-treated, de-*O*-acylated intact LPS indicated that 14:0(3-OH), 18:0(3-OH), and 16:0(3-OH) were base resistant and likely are the major amide-linked acyl substituents of Ko–lipid A. On the other hand, the straight-chain 16:0, 18:1, 18:0, 19:1, and 14:0(3-OH) were recovered in the chloroform extract from base-treated LPS; thus, it is likely these fatty acids are connected to GlcN through an ester bond.

**Figure 5:**
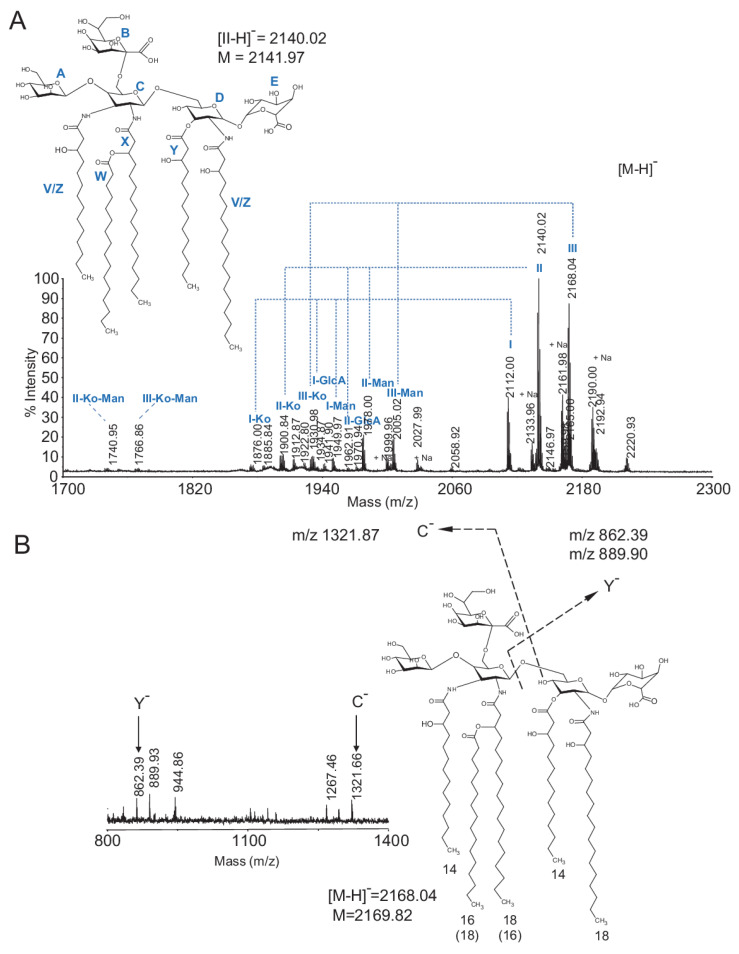
Matrix assisted laser desorption/ionization time of flight mass spectrometry (MALDI-TOF MS) analysis of *G. bethesdensis* Ko–lipid A. The spectrum was acquired in a negative ionization reflectron mode. (**A**) [M-H]- ions observed in the Ko–lipid A sample and proposed structure corresponding to the major ion at *m*/*z* 2140.02; (**B**) low-intensity Y^−^ and C^−^ fragment ions observed in the *m*/*z* 800–1400 range of the spectrum due to in-source cleavage of the parent *m*/*z* 2168.04. Legend: Structures I, II, II are due to lipid A having the same sugar backbone but substituted with acyl chains of different lengths; (-CH2-CH2-)_n_; for details, refer to Table 3; structures marked with -Man, -GlcA, and -Ko are due to the loss of Man (*m*/*z* 162) or GlcA (*m*/*z* 176) or Ko (*m*/*z* 236) from the main structure, respectively; A,B,C,D,E in bold blue: sugar residues identified in NMR analysis; V,W,X,W,Z in bold blue: possible assignments of acyl chain residues based on NMR analysis.

The presence of amide- and ester-linked fatty acids substituting the Ko–lipid A backbone was supported by NMR experiments (Figure 4 and Table 2). The TOCSY spectrum clearly showed four sets of α-methylene protons with correlations in the downfield region of the spectrum, indicative of 3-hydroxy fatty acids. Three of these sets (V, Y, and Z in Table 2 and Figure 4 and the structure inset in Figure 5A) correlated with signals around 4 ppm, a chemical shift that is consistent with that of H-3 of unsubstituted 3-hydroxy fatty acids. The fourth set (X in Table 2) showed cross peaks with a signal above 5 ppm, suggesting a 3-oxyacyl residue.

The chemical shifts of the α-protons of one of the unsubstituted fatty acids (Y in Table 2) were significantly more downfield than the other two, suggesting that this fatty acid was linked as an ester to O-3 of residue D, while the other two (V and Z in Table 2) were linked as amides to amino groups of GlcNAc or GlcN3N. A set of correlated peaks corresponding to the non-hydroxylated straight-chain fatty acids was also found (W in Table 2). It was not possible to precisely assign which amino group of the backbone was substituted by which of the amide-linked acyl chains (X, V, Z). In conclusion, *G. bethesdensis* Ko–lipid A is a penta-acylated molecule and its Glc*p*N3N-Glc*p*N backbone is substituted by three amide- and one ester-linked 3-hydroxy fatty acids. The fifth acyl residue is a straight-chain fatty acid (acyloxyacyl residue) that is ester-bound to the 3-hydroxy residue of the primary amide-linked fatty acid. The acyl chain length heterogeneity observed in the compositional analyses, the carbohydrate backbone structure, and the acylation types deduced both from NMR and composition studies were further supported by the results of MALDI-TOF MS.

MALDI-TOF MS analysis of intact Ko–lipid A in the negative ionization reflectron mode revealed the presence of two clusters of ions, each of which was composed of signals differing by 28 Da, corresponding to a difference in the fatty acid chain length (-CH_2_-CH_2_-), substituting lipid A (for MALDI-TOF MS details, refer to Figure 5A and Table 3). This finding was corroborated by the identification of 14:0(3-OH), 16:0(3-OH), 18:0(3-OH), 16:0, and 18:0 in chemical analyses. Based on NMR and compositional data, we propose that the major monoisotopic [M-H]^−^ ions were due to penta-acyl Ko–lipid A composed of a Man-GlcN3N(Ko)-GlcN-GlcA pentasaccharide substituted with either (14:0(3-OH))_3_, 16:0, 18:0(3-OH) at *m*/*z* 2112.00 (MALDI ion labeled with I) or (14:0(3-OH))_2_, 16:0(3-OH), 16:0, 18:0(3-OH) at *m*/*z* 2140.02 (MALDI ion labeled with II) or (14:0(3-OH))_2_, 16:0, (18:0(3-OH))_2_ at *m*/*z* 2168.04 (MALDI ion labeled with III).

Further spectral complexity arose from structures substituted with unsaturated fatty acids (−2 Da) and by a formation of sodium adducts in MS analysis. The small cluster of ions ranging from *m*/*z* 1876–2028 was due to loss of Man (*m*/*z* 162) or GlcA (*m*/*z* 176) or Ko (*m*/*z* 236) from the main structure (ions marked with -Man, -GlcA, and -Ko, respectively (Figure 5A and Table 3)) and were also observed in their sodiated forms. In addition, we observed two minor structures in which the penta-acyl lipid A was missing both Man and Ko residues (*m*/*z* 1740.95 and 1769.85) from the main structure (structures marked with -Ko-Man). The overall Ko–lipid A structure was further supported by the detection of minor MALDI-TOF MS signals due to in-source cleavage of Ko–lipid A via its β-GlcN3N-(1→6)-α-GlcN glycosidic bond, i.e., C^−^, Y^−^. This cleavage generated (from the native *m*/*z* 2168.04 ion) the C^−^ fragment at *m*/*z* 1321.87 due to Man-GlcN3N(Ko) disaccharide substituted with (14:0(3-OH), 18:0-3-O(16:0)) and the Y^−^ fragment at *m*/*z* 862.39 due to GlcN-GlcA disaccharide substituted with (14:0(3-OH), 18:0(3-OH)). There was a small 28 Da variation possibly caused by a difference in the acyl chain length (Figure 5B).

**Figure 6:**
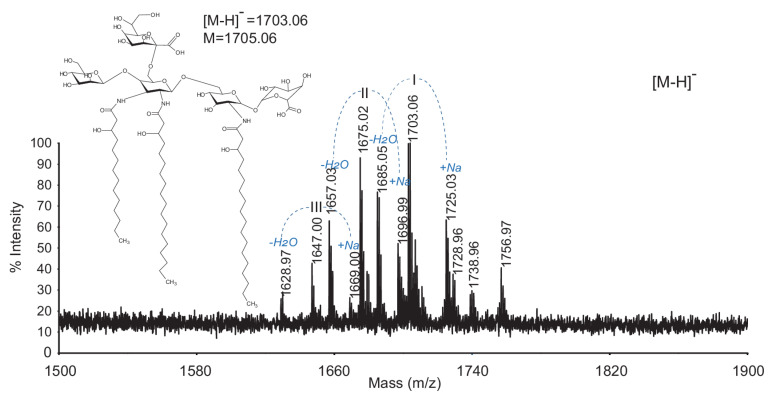
MALDI-TOF MS of de-*O*-acylated *G. bethesdensis* Ko–lipid A and the proposed acylation pattern after chemical degradation. The spectrum was acquired in a negative reflectron mode [M-H]^−^. For proposed compositions, refer to Table 4.

The structures of the ester-linked fatty acids present in *G. bethesdensis* Ko–lipid A were confirmed by MALDI-TOF MS after lipid A de-*O*-acylation. The spectrum acquired in the negative ionization reflectron mode displayed a cluster of [M-H] ions due to chemical cleavage and the loss of two acyl chains from the native penta-acyl-(Man-GlcN3N(Ko)-GlcN-GlcA) lipid A. Thus, the ion at *m*/*z* 1703.06 is consistent with the loss of the ester-linked 16:0 and 14:0(3-OH) fatty acids from the main structure (*m*/*z* 2168.04) but still containing the remaining (18:0(3-OH))_2_ and 14:0(3-OH) amide-linked fatty acids (Figure 6 and Table 4). The ion at *m*/*z* 1675.03 is consistent with the loss of 16:0 and 14:0(3-OH) from the penta-acyl Ko–lipid A (a loss from the native ion at *m*/*z* 2140.02) and still contains the 18:0(3-OH), 14:0(3-OH), and 16:0(3-OH) fatty acyl residues. Similarly, the ion at *m*/*z* 1647.00 is consistent with the loss of 16:0 and 14:0(3-OH) from the main penta-acyl Ko–lipid A (a loss from the native ion at *m*/*z* 2112.00), which is now only substituted with amide-linked 18:0(3-OH) and (14:0(3-OH))_2_. These observed ions were also accompanied with corresponding sodiated, and anhydro forms, respectively (for details, refer to Figure 5B and Table 4).

In conclusion, based on a combination of chemical and structural studies, we propose that *G. bethesdensis* synthesizes a heterogeneous Ko–lipid A built of an α-Man*p*-(1→4)-β-Glc*p*N3N-(1→6)-α-Glc*p*N-(1↔1)-α-Glc*p*A tetrasaccharide backbone substituted with five acyl groups varying in chain length, of which three are amide-linked primary β-hydroxylated fatty acids and one is an ester-linked primary β-hydroxylated fatty acid. In addition, one of the primary amide-linked fatty acids is substituted with a secondary straight-chain fatty acid via an ester bond. In particular, the β-Glc*p*N3N residue is substituted with 14:0(3-OH) and 16:0(3-O(16:0)) at its N-2 and N-3, and the Glc*p*N residue is substituted with 14:0(3-OH) and 18:0(3-OH) at its O-3 and N-2, respectively. The chain length of these acyl chains might differ. In addition, the Glc*p*N3N of lipid A is covalently substituted with an α-Ko-(2→6) residue that is highly resistant to acid hydrolysis.

### 2.5. Induction of Cytokine Production by Purified Granulibacter Ko–Lipid A in Human Whole Blood

**Figure 7:**
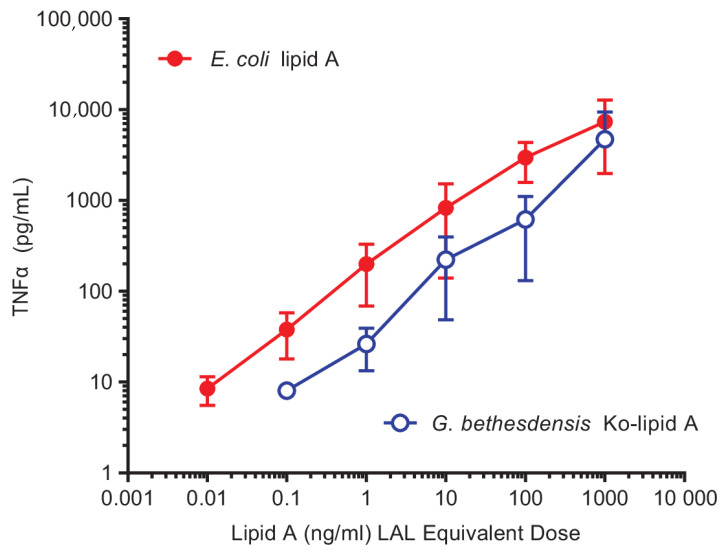
Induction of TNFα secretion in human blood ex vivo: *Limulus* amoebocyte lysate (LAL) equivalent doses of *G. bethesdensis* Ko–lipid A or *E. coli* lipid A were added to blood and incubated for 6 h, and the resulting supernatants were assayed for TNFα concentration by ELISA. Data shown represent the mean ± SD (*n* = 12 donors).

Intact *G. bethesdensis* bacterial cells were hypostimulatory compared to *E. coli* in purified human neutrophils [12], monocytes, and monocyte-derived macrophages [13] and in whole blood ex vivo (Figure 1). Comparison of lipid A from different species based on nominal mass was complicated by the presence of variable amounts of uncleaved intact LPS with variable amounts of polysaccharide. Although it remains to be shown whether *G. bethesdensis* Ko–lipid A activates the *Limulus* amoebocyte lysate (LAL) to the same extent as equal molar amounts of *E. coli* lipid A, we chose to use bioactivity, as measured in the LAL, as a point of comparison. LAL activity in samples was compared to an external standard LPS, and concentrations were expressed as LAL equivalent units. Venous blood from healthy subjects was anticoagulated with citrate and exposed to various concentrations of lipid A for 6 h at 37 °C, as described in the Material and Methods section, and the concentrations of TNFα were measured by ELISA. Similar to the results with intact bacteria, purified *G. bethesdensis* Ko–lipid A is approximately 10–100 times less potent than *E. coli* lipid A (Figure 7).

## 3. Discussion

In this study, we purified and characterized the Ko–lipid A glycolipid of *G. bethesdensis*, a pathogen in patients with CGD. As an intact bacterium, *G. bethesdensis* was 10–100 times less potent than *E. coli* as a stimulator of human polymorphonuclear leukocytes, as measured by NADPH oxidase activation [10]; monocytes and macrophages, as measured by cytokine production [11]; and total peripheral blood cells (Figure 1). This unusually poor stimulatory activity led us to characterize lipid A from the type strain of *G. bethesdensis.* We showed that the carbohydrate backbone of this glycolipid consists of an α-Man*p*-(1→4)-β-Glc*p*N3N-(1→6)-α-Glc*p*N-(1↔1)-α-Glc*p*A tetra-saccharide in which the (-β-Glc*p*N3N-(1→6)-α-Glc*p*N-) disaccharide component is substituted with five acyl chains, including amide-linked N-3′ 14:0(3-OH) and N-2′ 16:0(3-O16:0), and ester-linked O-3 14:0(3-OH) and 16:0. These fatty acid substituents can differ in chain length and vary between 14:0(3-OH) and 18:0(3-OH) or 16:0/18:0 by (-CH_2_-CH_2_-) mass increments, contributing to structural microheterogeneity. In addition, C-6 of Glc*p*N3N is substituted with a rarely occurring α-Ko-(2→6)-α-d-*glycero*-d-*talo*-oct-2-ulosonic acid residue resistant to acid hydrolysis. The (-β-Glc*p*N3N-(1→6)-α-Glc*p*N-) component of this Ko–lipid A has also been called hybrid lipid A since it consists of a mixture Glc*p*N3N and Glc*p*N rather than either two Glc*p*N3N or two Glc*p*N residues [25]. Thus far, this type of lipid A has been reported in *Campylobacter jejuni* [25] and *Acetobacter pasteurianus* [18,19]. In addition, this hybrid disaccharide Ko–lipid A component is unusual in that it is substituted with an acidic α-(1↔1)-α-Glc*p*A residue at the proximal Glc*p*N residue and with a Man*p* residue at the C-4′ of the distal Glc*p*N3N residue. Lipid A from enteric bacterial species generally has a *bis*-phosphorylated Glc*p*N-Glc*p*N disaccharide backbone, with the phosphate groups providing the negative charges. In this case, one of the phosphates is replaced by a neutral sugar, Man*p*, and the other by the less anionic carboxyl group of Glc*p*A. These structural differences may contribute to *G. bethesdensis* resistance to cationic antimicrobial peptides such as the human LL-37 cathelicidin peptide [12]. The Ko–lipid A backbone of *G. bethesdensis* we describe is similar to that recently reported for *Acetobacter pasteurianus*; however, the lipid A described from *A. pasteurianus* differs significantly in its fatty acylation pattern compared to *G. bethesdensis* Ko–lipid A in that *G. bethedensis*’s is penta-acylated, whereas that of *A. pasteurianus* is hexa-acyclated [18,19].

Other pathogens and opportunistic pathogens have Ko in their lipid A, including *Acinetobacter calcoaceticus* [23] and some species of *Yersina* and *Burkholderia* [26]. Within *Alphaproteobacteria,* to which *Acetobacteraceae* belong, *Brevundimonas diminuta* (formerly *Pseudomonas diminuta*) [27], has been reported to have Ko, although other species have Kdo instead, for example, *Bartonella henselae* [28]. *Bartonella quintana* lipid A is an antagonist of TLR4 but appears to possess Kdo [29]. Furthermore, Ko has been reported to be an inducible modification, which may help explain why even within the same genus (e.g., *Acetobacter*), some species appear to have Kdo (*A. methanolica*) and others Ko (*A. pasteurianus*). Further work will be required to determine whether inducible alterations in *G. bethesdensis* Ko–lipid A occur and whether locking a strain genetically into particular biosynthetic options impacts virulence in animals. Importantly, the genome of *G. bethesdensis* does not contain an obvious homologue of KdsC, the 3-deoxy-D-*manno*-ulosonate 8-phosphate phosphatase that is required for production of Kdo [30], and we were unable to detect Kdo in any of the *G. bethesdensis* LPS or Ko–lipid A preparations tested.

2,3-Diamino-2,3-dideoxy-d-glucose (GlcN3N)-containing lipid A backbones occur in a variety of pathogenic and non-pathogenic species (e.g., *Aquifex pyrophilus, Bartonella henselae, Brucella abortus, Bacteriovorax stolpii, Bradyrhizobium elkanii, Caulobacter crescentus, Legionella pneumophila, Mesorhizobium huakuii,* and *Mesorhizobium loti*). Moreover, there is a great diversity in the presence of either Glc*p*N or Glc*p*N3N in the lipid A backbones [31]. The biosynthesis of Glc*p*N3N in *Acidithiobacillus ferrooxidans* requires the two-step conversion of a uridine diphosphate glucose (UDP)-GlcNAc precursor by GnnA oxidoreductase and GnnB transaminase [32]. It has been shown that inactivation of *gnnA* and *gnnB* in *Campylobacter jejuni* results in an increase in the otherwise low activation of TLR4 and an increase in the sensitivity of *C. jejuni* to antimicrobial peptides [33]. In *G. bethesdensis*, possible orthologues of these genes include one copy of a putative *gnnA* gene (ABI62343.1, Nicotinamide adenine dinucleotide (NAD)-dependent oxidoreductase) with 44% amino acid identity to *gnnA* from *Acidithiobacillus ferrooxidans* and two possible *gnnB* genes (ABI62037.1 and ABI61062.1) with 37% and 39% amino acid identity, respectively, to *gnnB*. Genetic studies are now underway to determine whether *gnnA* and *gnnB* in *G. bethesdensis* are responsible for Glc*p*N3N formation, poor TLR4 activation, and high intrinsic resistance to cationic antimicrobial peptides [12]. Rather than being a constant structure, lipid A can undergo inducible modifications as microbes adapt to changing environments, including that within the phagosome [34,35,36,37]. At present, we do not know whether the structure described herein is the same or different from that occurring during growth in vivo. Indeed, the expression of some of the above-mentioned *gnnA* and *gnnB* genes putatively involved in Glc*p*N3N biosynthesis was altered during intracellular growth of *G. bethesdensis* within experimentally infected human leukocytes [38], raising the possibility that *G. bethesdensis* lipid A may vary under different conditions.

The biosynthesis of lipid A in which the disaccharide carbohydrate backbone is not substituted with phosphates at the 1′- and 4′-ends but is instead modified with other carbohydrate substituents requires the activity of 1′-LpxE- and 4′-LpxF-specific phosphatases, respectively [39,40]. *G. bethesdensis* contains an LpxE-like phosphatase (WP_011632924) with similarity to that reported in *R. leguminosarum* bv. viciae 3841 (RL4708, CAK10191.1), *Rhizobium etli*, as well as *R. leguminosarum* (RL1570) [41]. Although it remains to be proven biochemically, the presence of these homologues is consistent with the carbohydrate substitutions we detected.

In previous work on the lipid A structure from *M. loti* MAFF303099, it was proposed that the *rgtF* gene encoding for the Mlr0011 protein (BAB47689.1) of the ArnT superfamily is a putative α-(1-1)-GalA transferase [24]. The *G. bethesdensis* genome contains a gene, WP_011632457.1, with over 38% identity to mlr0011. Glycosyl transferases of the *arnT*-like type decorate the phosphates on the lipid A backbone with 4-amino-l-arabinose, thereby decreasing the local net negative charge in much the same way as phosphate does on prototypic enterobacterial lipid A. Although genetic deletion of *arnT* does not alter TLR4-stimulating activities of LPS, it decreases the resistance of bacteria to polymyxin B and other antimicrobial peptides and lowers the transmission of *B. bronchiseptica* from one host to another [37]. Failure to detect phosphate in the Ko–lipid A of *G. bethesdensis* may indicate that it is blocked, for example, through *arnT* activity. Further studies may be required to prove whether this modification plays an important part in the resistance of this bacterium to LL-37, the human cathelicidin peptide [12].

Although we have shown that the structure of the glycosyl portion of *G. bethesdensis* Ko–lipid A is identical with that of another member of *Acetobacteraceae*, namely *Acetobacter pasteurianus*, it appears that lipid A in this family is highly heterogeneous. The LPS of *Acidiphilium* GS18h, a soil isolate, contains Kdo and a GlcN backbone but is relatively non-toxic compared to the *E. coli* LPS [14,15]. The resistance of *Granulibacter* LPS to mild acid hydrolysis is similar to what has been reported for *Acetobacter methanolicus* [42], although the *A. methanolicus* LPS contains Kdo instead of Ko [42]. Interestingly, *A. methanolicus* is also capable of infecting patients with CGD [2], so this moiety, in and of itself, is not an absolute determinant of virulence in the setting of CGD. The LPS from *Acetobacter diazotrophicus* PAL5 was reportedly resistant to hydrolysis with 1% HOAc [43]. Resistance to acetic acid may not be surprising, since many members of this family produce acetic acid and can survive for years at HOAc concentrations in the 5–10% range (~1M HOAc). Clearly, Ko is not the only moiety conferring some degree of acid resistance, but acid resistance may be an important trait in a pathogen that may persist in acidified vacuoles [11,13].

The bulk of the purified LPS of *G. bethesdensis* appeared to consist of a polymer of rhamnose and glucose. Whether this represents an O-polysaccharide or a tightly associated capsular polysaccharide remains to be determined. A pentasaccharide repeat for the *Acetobacter diazotrophicus* O-polysaccharide has been identified to consist of one β-d-Glc, one β-d-Rib*f*, and three α-l-Rha residues [43].

*G. bethesdensis* Ko–lipid A is, like intact *G. bethesdensis* cells, significantly less stimulatory to human immune cells than is *E. coli* or its lipid A. Whether the unusual structure of its Ko–lipid A contributes to its pathogenicity and, possibly, to its ability to persist in a clinically silent state in vivo [10,44] will require the development of tools to genetically modify *G. bethesdensis* and further study.

## 4. Materials and Methods

### 4.1. Bacteria

The type strain of *G. bethesdensis* NIH1.1 (available from the American Type Culture Collection (ATCC, Manassas, VA, USA) as BAA-1260), originally isolated from a patient with CGD [4], was used for this study. For small-scale cultures, bacteria were grown in 2 L baffled flasks at 37 °C in yeast peptone glucose medium (YPG) [12], harvested by centrifugation, and inactivated, where noted, with alcohol after washing in saline. For large-scale cultures, a slightly altered composition of YPG was used (2% peptone, 1% yeast extract, 1% glucose), and 100 mL was inoculated with a frozen vial of cryopreserved bacteria and grown at 35 °C with shaking at 200 rpm for 2 days. This culture was inoculated into 1 L of YPG in a baffled flask and cultured for 1 day before transferring into 10 L in a 14 L fermenter. After growth for 48 h at 35 °C with O_2_ maintained at 50% air saturation (from the top), to an OD of 1.5, the culture was inactivated (with either 1% formalin or phenol) for 24 h prior to harvesting with a continuous-flow centrifuge.

### 4.2. Extraction and Purification of LPS

Cells were washed in phosphate-buffered saline (PBS) and in water and extracted three times with hot phenol-water [21]. The water, phenol, and interphase fractions were dialyzed (12–14 kDa cutoff), freeze-dried, then washed in 90% EtOH to reduce phospholipid contamination. Treatment with DNase, RNase, and proteinase K was followed by additional dialysis in 3.5-kDa-cutoff membranes at 4 °C for 4 days against deionized water. The samples were resuspended in water and centrifuged at 100,000× *g* for 20 h at 4 °C. The LPS was further fractionated either by gel filtration in the presence of deoxycholate, as previously described [45], or by direct analysis.

### 4.3. Isolation of Gb Ko–Lipid A Glycolipid

Mild acetic acid hydrolysis typically used to release lipid A from the LPS (1% HOAc at 100 °C) was not efficient even after 6 h. Harsher conditions employing 0.1 M HCl at 100–102 °C for 4.5 h resulted in more effective hydrolysis. The resulting colloidal hydrolysate was centrifuged at 5000× *g* at room temperature for 20 min. The supernatant was removed, and the pellet was washed two more times with water to reduce contamination with free polysaccharides or the unhydrolyzed LPS. The pellet was freeze-dried and further extracted with the Bligh–Dyer solvent system consisting of chloroform:methanol:water (2:2:1.8; *v*/*v*/*v*). The organic bottom phase was removed, extracted back with water, collected, and used for further studies.

### 4.4. Compositional Analysis of LPS

Compositional analysis was performed using trimethylsilyl (TMS) methylglycoside derivatives after 18–20 h of methanolysis with 1–2 M HCl-MeOH at 80 °C, as previously described [46,47]. Fatty acid methyl esters (FAMEs) were similarly prepared; optionally, total FAMEs were prepared after hydrolysis of the sample with 4 N HCl and conversion to TMS-methyl esters, as previously described [24].

The 2,3-diamino-2,3-dideoxy-D-glucose (Glc*p*N3N) in lipid A was determined after hydrolysis of lipid A with 4 M HCl for 4 h at 121 °C, followed by peracetylation, reduction with NaBD_4_, and conversion to alditol acetates. The GlcpN3N was assigned based on characteristic GC-EI mass fragmentation of the alditol derivative [24,48]. All chemical analyses were performed on a Hewlett-Packard HP5890 gas chromatograph equipped with a mass selective detector (5970 MSD) and an Alltech AT-1 fused silica capillary column (30 m × 0.25 mm I.D.) with helium carrier gas.

The linkage analysis of neutral and acidic sugars was done by preparing partially methylated alditol acetates (PMAAs) after mild methyl carboxyl esterification and carboxyl group reduction prior to permethylation and classical steps of linkage analysis, as previously described [49].

### 4.5. Analysis of O-deacylated Lipid A

Ester-linked fatty acids were released from the LPS by 20 min incubation in 1 M NaOH at 80 °C. The reaction was quenched with 1 M HCl, and free fatty acids were extracted with chloroform and used for chemical analysis. The remaining de-*O*-acylated LPS was dialyzed (1000 MWCO) against H_2_O, freeze-dried, and hydrolyzed to liberate *O*-deacylated lipid A. The lipid A was further purified, as described in a previous section, and used for fatty acid composition and matrix-assisted laser desorption/ionization time of flight mass spectrometry (MALDI-TOF MS) studies.

### 4.6. MS Analysis of Lipid A

Lipid A samples were suspended in 2:1 chloroform:methanol (*v*/*v*) and mixed in 1:1 (*v*/*v*) ratio with 0.5 M 2,4,6-trihydroxyacetophenone in MeOH and spotted onto a MALDI plate. MALDI–time-of-flight (TOF) MS and MS/MS spectra were acquired on the Applied Biosystems AB SCIEX TOF/TOF 5800 system in the negative or positive ionization reflectron detection mode and processed with AB Data Explorer.

### 4.7. Nuclear Magnetic Resonance (NMR) Spectroscopy

To probe the structure of *Granulibacter* lipid A, 1D ^1^H (proton) and 2D ^1^H-^1^H homonuclear correlation spectroscopy (COSY), 2D ^1^H-^1^H total correlation spectroscopy (TOCSY), 2D ^1^H-^1^H nuclear Overhauser effect spectroscopy (NOESY), and ^1^H-^13^C heteronuclear single-quantum correlation spectroscopy (HSQC) experiments were performed at 30 °C on a Varian 600 MHz spectrometer equipped with a cold probe and processed with MestReC/nova software ver. 12.0.3-21384 (Mestrelab Research, Santiago de Compostela, Spain). Lipid A samples were dissolved in 3:1 chloroform-d:methanol-d_4_ (*v*/*v*). The COSY experiment was recorded using a set of 1024 increments with 16 scans, an acquisition time of 0.2 s, and 1 s saturation delay. The TOCSY experiment was recorded by using 256 increments with 32 scans, 80 ms spinlock time, and 0.15 s acquisition time. The NOESY experiment was recorded using a set of 256 increments with 16 scans, 200 ms mixing time, and 0.4 s acquisition time. The HSQC experiment was recorded using 128 increments with 96 scans and an acquisition time of 0.2 s.

### 4.8. Gel Electrophoresis and Staining

The LPS was resolved in PAGE by using 18% acrylamide and deoxycholic acid (DOC) detergent [50]. Resolved gels were silver-stained using the Bio-Rad Silver Staining Kit (Bio-Rad, Hercules, CA, USA). Optionally, the gels were stained with Alcian blue [51], followed by the same steps as with standard, regular silver staining.

### 4.9. Endotoxin Measurement

A kinetic chromogenic *Limulus* amoebocyte lysate assay (Chromo-LAL; Associates of Cape Cod, East Falmouth, MA, USA) was performed following the manufacturer’s instructions using a World Health Organization-traceable *E. coli* O113:H10 LPS standard for quantitation.

### 4.10. Whole-Blood Cytokine Assay

Venous blood was drawn after informed consent and anticoagulated with acid citrate dextrose. Assays were performed in Corning Costar 3596 plates and included 90 µL of anticoagulated blood and 10 µL of stimulus or buffer controls. After 6.5 h incubation at 37 °C in 5% CO_2_, 200 µL of ice-cold RPMI 1640 was mixed into the samples and the plate centrifuged for 15 min at 3000× *g* at 4 °C. Supernatants were transferred to a polypropylene microplate and stored at −80 °C. TNFα was measured by ELISA (eBiosciences, San Diego, CA, USA) as per the manufacturer’s instructions.

### 4.11. Statistics

Data analysis was performed using Prism version 6 (GraphPad Software, San Diego, CA, USA).

## Figures and Tables

**Table 1:** NMR assignments of glycosyl residues identified in the Ko–lipid A of *G. bethesdensis*. For NMR spectra, refer to Figure 3.

No.	Residue	Chemical Shift (ppm)							
		1	2	3	4	5	6	7	8
D	6-α-GlcN	5.06	4.26	5.16	3.41	4.10	4.05/3.58		
		93.1	51.1	74.3	69.0	71.4	68.3		
E	α-GlcA	5.13	3.65	3.85	3.65	4.17			
		94.3	71.1	72.3	72.0	71.5	ND		
A	α-Man	4.96	3.74	3.56	3.61	3.43	3.63/3.60		
		101.8	70.7	70.7	67.4	73.9	64.1		
C	4,6-β-GlcN3N	4.36	3.70	4.03	3.42	3.60	3.85/3.80		
		101.4	53.4	54.7	75.8	74.3	63.1		
B	α-Ko			3.98	3.85	4.09	3.77	4.03	3.81/3.69
		ND	ND	71.6	65.9	68.8	71.8	70.1	61.8

Legend: GlcN, glucosamine; GlcN3N, 2,3-diamino-2,3-dideoxy glucose; Ko–d-*glycero*-d-*talo*-oct-2-ulosonic acid; all residues are in *p* configuration; ND, not determined.

**Table 2:** NMR assignments of fatty acid substituents in the Ko–lipid A of *G. bethesdensis*. For NMR spectra, refer to Figure 3 and Figure 4.

Acyl	Residue	Chemical Shift (ppm)				
Chain No.		2	3	4		
V	(3-OH)-FA	2.33/2.26	3.92	1.44/1.39		
	amide linked	42.9	68.5	37.4		
W	FA	2.27/2.27	1.58/1.58	1.27/1.27		
	ester linked	34.5	25.1	29.6		
X	(3-OR)-FA	2.45/2.31	5.07	1.52/1.52		
	amide linked	41.2	71.0	34.0		
Y	(3-OH)-FA	2.49/2.39	3.98	1.48/1.41		
	ester linked	42.2	68.5	37.2		
Z	(3-OH)-FA	2.31/2.18	3.97	1.41/1.41		
	amide linked	44.2	68.5	37.5		
		bulk -CH_2_-	ω-3	ω-2	ω-1	ω
	All fatty acids	1.27/1.27	1.38/1.29	1.24/1.24	1.27/1.27	0.87
		29.6	25.5	31.9	22.6	14.0

Legend: 3-OH-FA, 3-hydroxy fatty acid; FA, straight-chain fatty acid; (3-OR)-FA, 3-hydroxy-substituted fatty acid.

**Table 3:** Major [M-H]^−^ molecular ions observed in MALDI-TOF MS analysis of *G. bethesdensis* Ko–lipid A and proposed composition (corresponding MALDI-TOF MS spectrum in Figure 5A).

Ion, Peak Label	Obs. [M-H]^−^	Predicted [M-H]^−^	MW [M]	Glycosyl Composition	Proposed FA Composition (penta-acyl Lipid A)
I	2112.00	2111.36	2113.72	Man, GlcN3N(Ko), GlcN, GlcA	(14:0(3-OH))_3_; 16:0; 18:0(3-OH)
II	2140.02	2139.39	2141.79	Man, GlcN3N(Ko), GlcN, GlcA	(14:0(3-OH))_2_; 16:0(3-OH); 16:0; 18:0(3-OH)
III	2168.04	2167.42	2169.82	Man, GlcN3N(Ko), GlcN, GlcA	(14:0(3-OH))_2_; 16:0; 18:0(3-OH)_2_
I-GlcA	1934.87	1935.33	1937.59	Man, GlcN3N(Ko), GlcN	(14:0(3-OH))_3_; 16:0; 18:0(3-OH)
II-GlcA	1963.91	1963.36	1965.65	Man, GlcN3N(Ko), GlcN	(14:0(3-OH))_2_; 16:0(3-OH); 16:0; 18:0(3-OH)
I-Man	1949.97	1949.30	1951.58	GlcN3N(Ko), GlcN, GlcA	(14:0(3-OH))_3_; 16:0; 18:0(3-OH)
II-Man	1978.00	1977.34	1979.63	GlcN3N(Ko), GlcN, GlcA	(14:0(3-OH))_2_; 16:0(3-OH); 16:0; 18:0(3-OH)
III-Man	2005.02	2005.37	2007.68	GlcN3N(Ko), GlcN, GlcA	(14:0(3-OH))_2_; 16:0; 18:0(3-OH)_2_
I-Ko	1876.00	1875.31	1877.54	Man, GlcN3N(Ko), GlcN, GlcA	(14:0(3-OH))_3_; 16:0; 18:0(3-OH)
II-Ko	1902.95	1903.34	1905.59	Man, GlcN3N(Ko), GlcN, GlcA	(14:0(3-OH))_2_; 16:0(3-OH); 16:0; 18:0(3-OH)
III-Ko	1930.98	1931.37	1933.65	Man, GlcN3N(Ko), GlcN, GlcA	(14:0(3-OH))_2_; 16:0; 18:0(3-OH)_2_
II-Ko-Man	1740.95	1741.28	1743.45	GlcN3N(Ko), GlcN, GlcA	(14:0(3-OH))_2_; 16:0(3-OH); 16:0; 18:0(3-OH)
II-Ko-Man	1769.85	1769.31	1771.50	GlcN3N(Ko), GlcN, GlcA	(14:0(3-OH))_2_; 16:0; 18:0(3-OH)_2_

Legend: 3-OH-FA, 3-hydroxy fatty acid; FA, straight-chain fatty acid; (3-OR)-FA, 3-hydroxy-substituted fatty acid; Obs., observed *m*/*z* in MS analysis; MW, molecular weight.

**Table 4:** Major [M-H)]^−^ ions observed in the MALDI-TOF MS analysis of de-*O*-acylated *G. bethesdensis* Ko–lipid A and proposed compositions (corresponding MALDI-TOF MS spectrum in Figure 6).

Ion	Obs. [M-H]^−^	Predicted [M-H]^−^	MW [M]	Glycosyl Composition	Proposed FA Composition de-*O*-acylated (Triacyl-Lipid A)
I	1703.06	1703.00	1705.06	Man, GlcN3N(Ko), GlcN, GlcA	18:0(3-OH); 16:0(3-OH); 14:0(3-OH)
	1725.02	1725.99	1728.05	(Man, GlcN3N(Ko), GlcN, GlcA)Na	18:0(3-OH); 16:0(3-OH); 14:0(3-OH)
	1685.05	1684.99	1687.04	(Man, GlcN3N(Ko), GlcN, GlcA)anhydro	18:0(3-OH); 16:0(3-OH); 14:0(3-OH)
II	1675.03	1674.97	1677.01	Man, GlcN3N(Ko), GlcN, GlcA	18:0(3-OH); 16:0(3-OH); 14:0(3-OH)
	1696.99	1697.96	1699.99	(Man, GlcN3N(Ko), GlcN, GlcA)Na	18:0(3-OH); 16:0(3-OH); 14:0(3-OH)
	1657.02	1656.96	1658.99	(Man, GlcN3N(Ko), GlcN, GlcA)anhydro	18:0(3-OH); 16:0(3-OH); 14:0(3-OH)
III	1647.00	1646.94	1648.95	Man, GlcN3N(Ko), GlcN, GlcA	18:0(3-OH); 14:0(3-OH)
	1669.99	1699.93	1671.94	(Man, GlcN3N(Ko), GlcN, GlcA)Na	18:0(3-OH); 14:0(3-OH)
	1628.97	1628.92	1630.93	(Man, GlcN3N(Ko), GlcN, GlcA)anhydro	18:0(3-OH); 14:0(3-OH)

Legend: FA, fatty acid; Obs., observed *m*/*z* in MS analysis; MW, molecular weight.
